# Troika of Posture, Occlusion and Airway

**DOI:** 10.1007/s12070-019-01734-7

**Published:** 2019-09-11

**Authors:** Sanghmitra Dasgupta, Joe E. Rozario

**Affiliations:** Bangalore, India

**Keywords:** Spine, Forward head syndrome, Posture, Occlusion, Airway, Sleep apnea, Snoring, Tinnitus, Impaired digestion, Weight gain

## Abstract

Good posture means spine is neutral. Posture is dictated by the existing occlusion, especially the relation between the maxillary and mandibular permanent first molars. Overloading the spine due to bad posture is the precursor of many ailments. Obstructive Sleep Apnea Syndrome is one of them. Compromised airway is found to be a major contributory factor in sleep apnea cases. Restoring the dental anatomy to its normal has been found to reduce the load on the spine, improving ones posture. It also facilitates the compromised airways to attain normalcy.

Spine or Merudanda as it is called in Sanskrit, is the name derived from sacred Mount Meru. Mt. Meru is considered as the axis of Earth. It is said the entire solar system revolves around it. Likewise spine is the axis of our body. Spine is the communication center between the brain and the rest of the body. All our emotions, feelings, movements, experiences, functioning of our every organ and even the act of breathing are routed through our Spine. If there is no spine, one would not have any of these experiences of his or her existence. That is why in higher level of consciousness, spine is also called as Axis of Universe.

Spine, the source of all our energy, is the first to form in mother’s womb. Spinal cord, a bundle of nerves, begins at the base of the brain and ends little below the level of rib cage. In the womb or prenatal period and following birth the shape of the spine in a baby is in the form of the letter ‘C’. This curve is known as primary curve. The primary curve is kyphotic. As long as the body is prone, the body weight does not shift to the spine. When the baby starts to walk, the muscles begin to develop and once the muscle strength is adequate, the body weight shifts to the spine. The secondary curves form and are Lordotic. These curves continue to develop till growth spurts exist in the child.

Since spinal chord housed in the vertebral column support the head, once the secondary curves are formed head positioning, relationship of mandible with maxilla and relationship of mandibular teeth with maxillary teeth play a role in stabilization of cervical spine.

For spine to remain neutral core muscles play the role of stabilizer. Along with core muscles articulation of mandible with maxilla and relationship of mandibular teeth with maxillary teeth in three dimensions dictate posture. If the relationship is normal, spine remains neutral.

Posture is defined as an optimal body position where in an individual holds the body standing, sitting or lying down or working in such a way that the spine remains neutral. If the spine is neutral it is termed as good posture (Fig. 1).

**Fig. 1:**
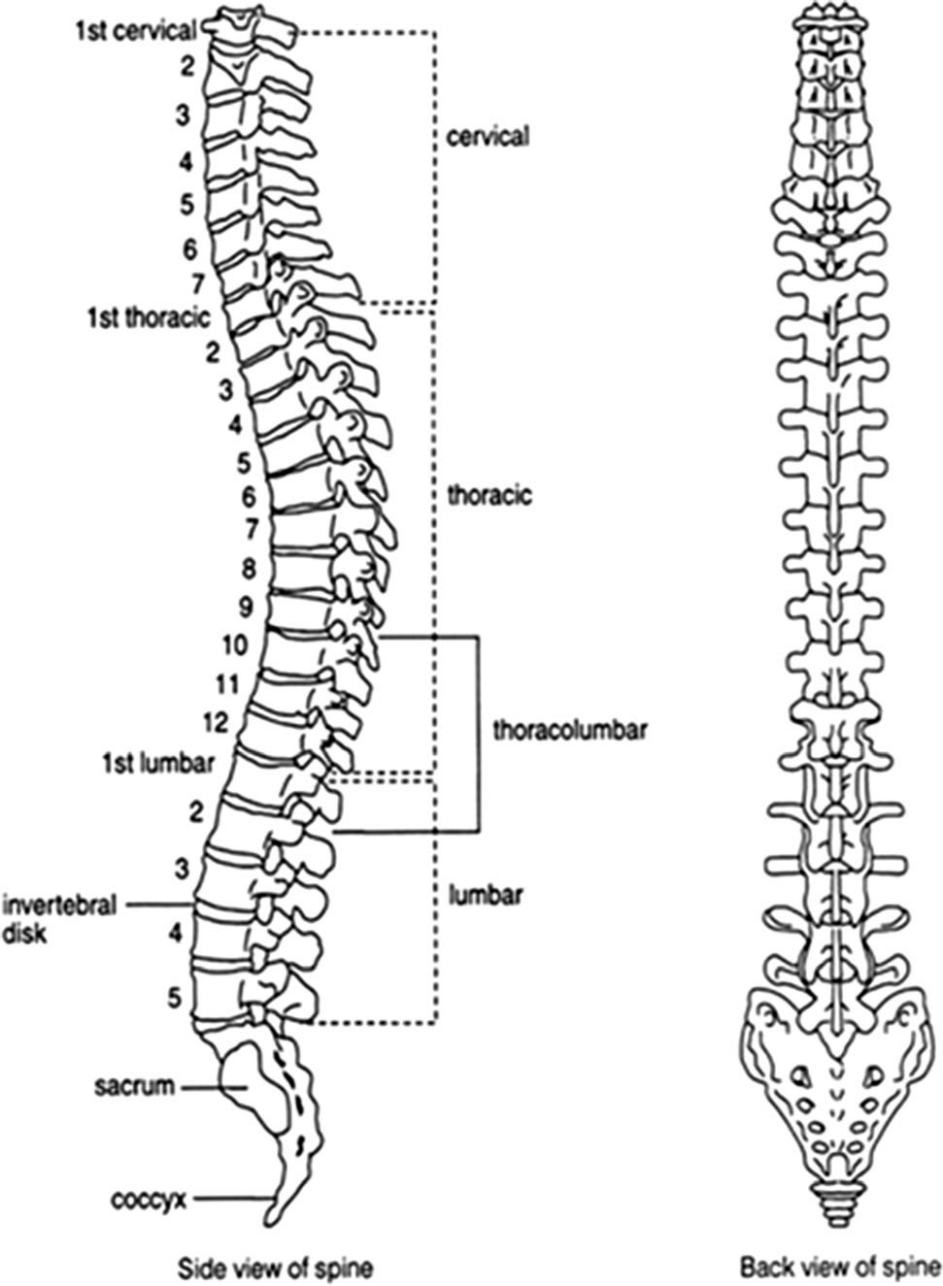
Normal spine

Spine can withstand only 4–4.5 k of load. If the spine is not neutral and being subjected to more load than what it can withstand, spine begins to degenerate. That is the start of onset of diseases.

Spine formation in a child is completed by 5–6 years. Growing spine (secondary curves) now requires stability. It is provided by core muscles and relationship of mandible with maxilla and the teeth size, angle and height of first molars in particular (Fig. 2).

**Fig. 2:**
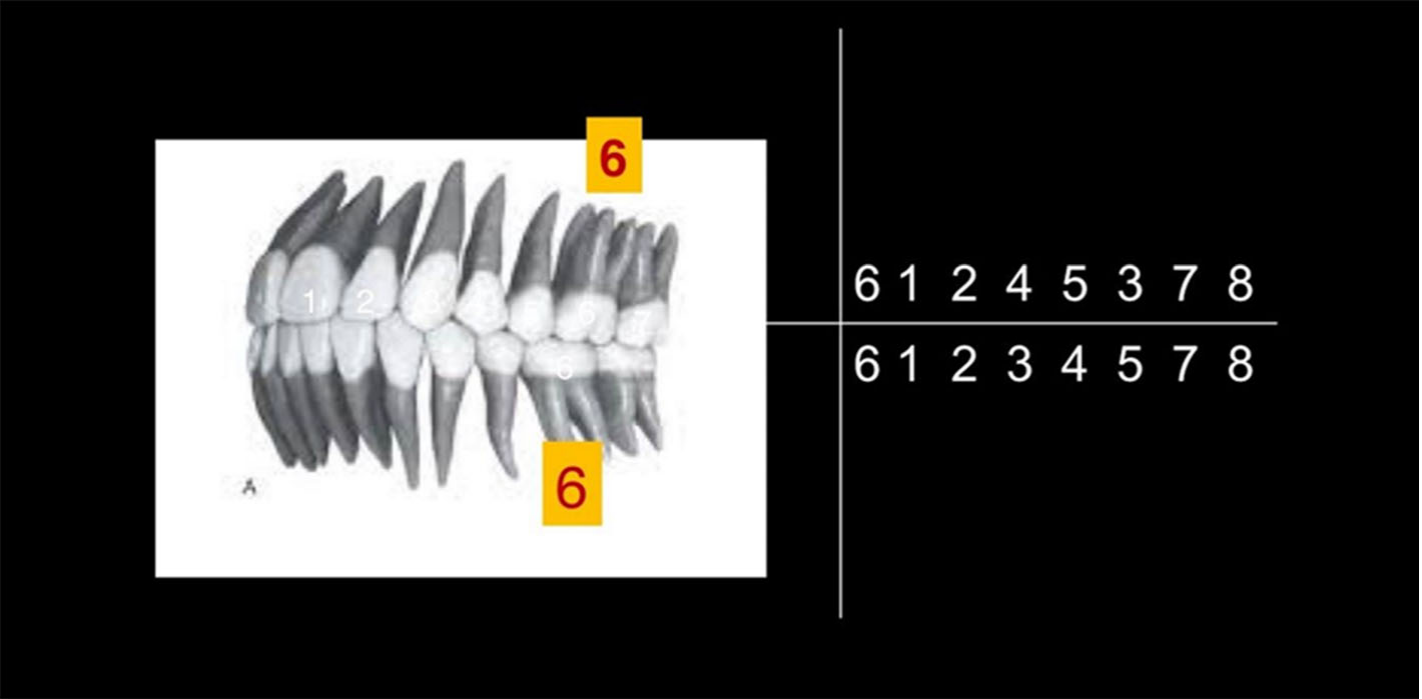
Sequence of eruption of permanent teeth

At the age of 6–7 years first molar tooth appears to give that stability to the cervical spine. First molars are the teeth with biggest surface area, and they can withstand maximum biting force. Optimal height and relationship ensures that cervical spine remains in most neutral position. In this position head generates about 4.5–5 k of force on the spine, which it can withstand with ease and with least amount of energy expenditure.

The first molar relationship between the maxillary and mandibular jaws dictate posture. Changes in the position of the mandible influence body posture. Reciprocally body posture seems to have an effect on the position of the mandible [1].

In the year 1899 Edward H. Angle published the first classification on malocclusion based on the permanent first molar relationship (Figs. 3, 4, 5).

**Fig. 3:**
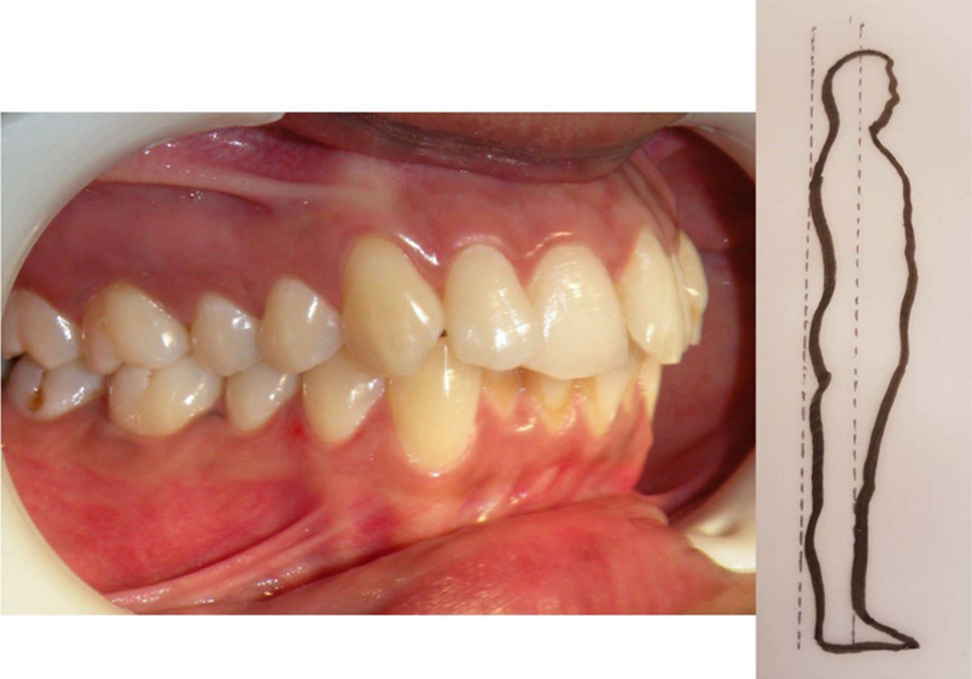
Angles Class I relationship between mandibular first molar and maxillary first molar bilaterally. This is a good posture–straight posture

**Fig. 4:**
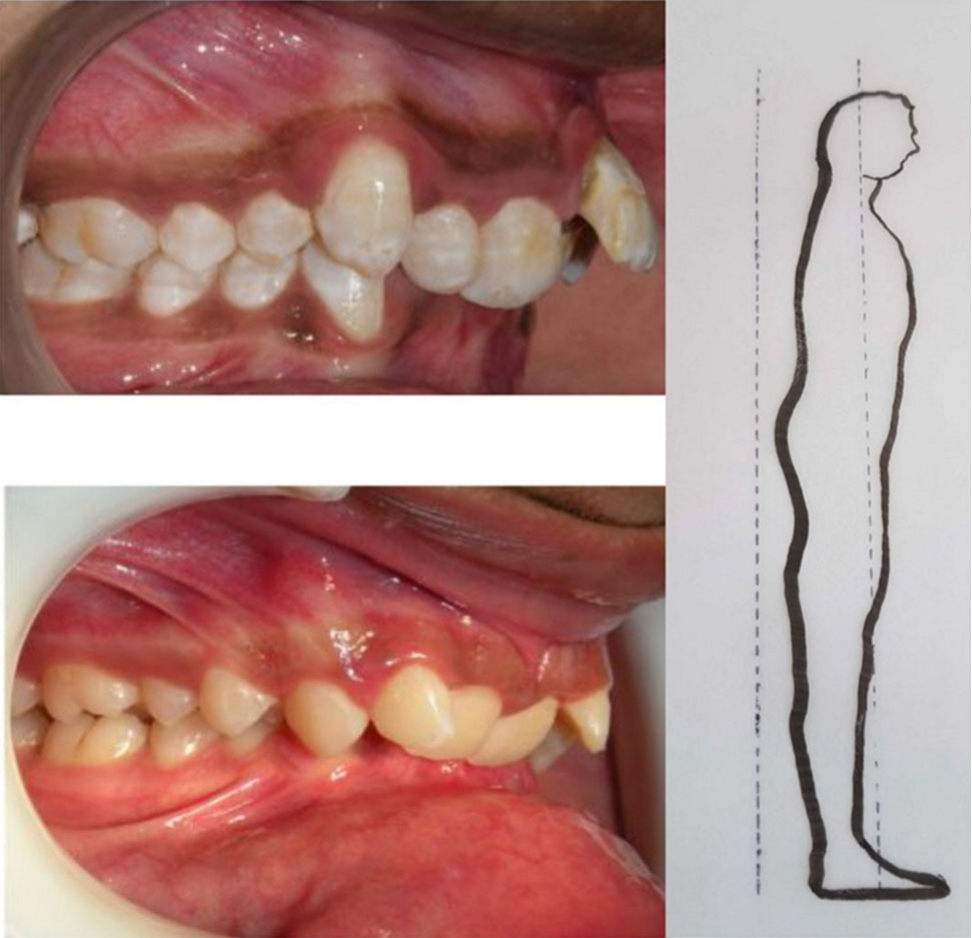
If mandible is behind maxilla and mandibular first molar tooth is behind the maxillary first molar tooth, irrespective of how the front teeth are, it is Angles Class II classification. Head falls forward termed as Kyphosis

**Fig. 5:**
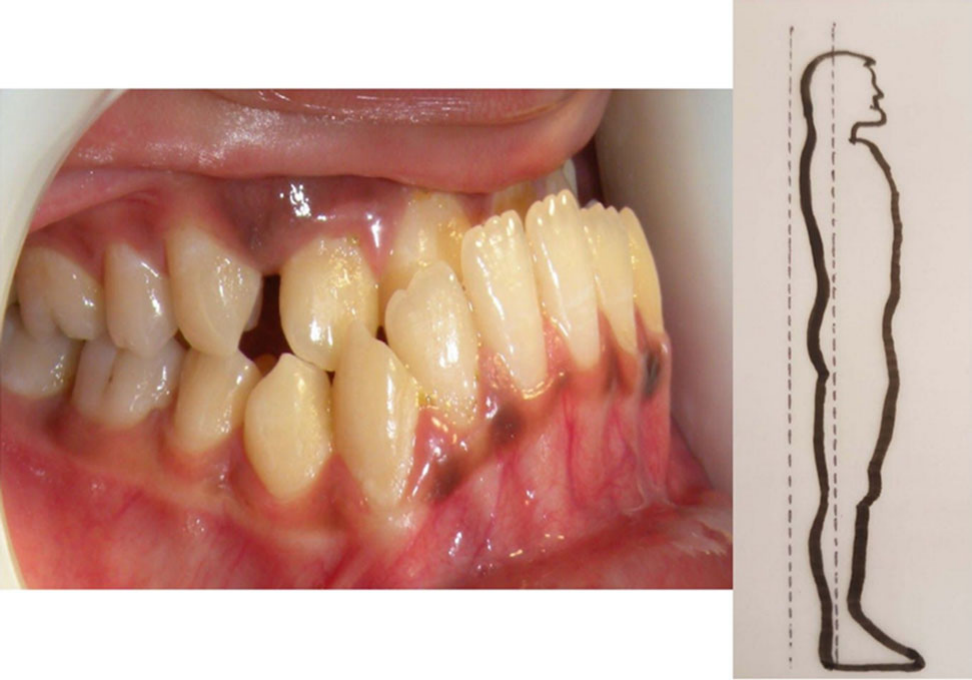
If the mandible is way ahead of maxilla and the mandibular first molar tooth is ahead of maxillary first molar it is Angles Class III classification. Head falls backwards termed as Lordosis

Rocabado et al. stated that there is an association between Class ll and Class lll occlusion and forward head posture [2]. It has been authenticated that subjects with Class ll molar occlusion had forward head posture and those with Class lll molar occlusion had backward head posture [1, 3]. Likewise the head posture can also influence the occlusion [4]. An association between head posture and the development of malocclusion was proposed by Schwartz way back in 1926 [5].

If the mandibular molars meet the maxillary molars in such a way that one side is higher than the other side the head is likely to be tilted to one side. This results in a cant of the maxilla leading to the body posture condition called Scoliosis (Figs. 6, 7, 8) [6].

**Fig. 6:**
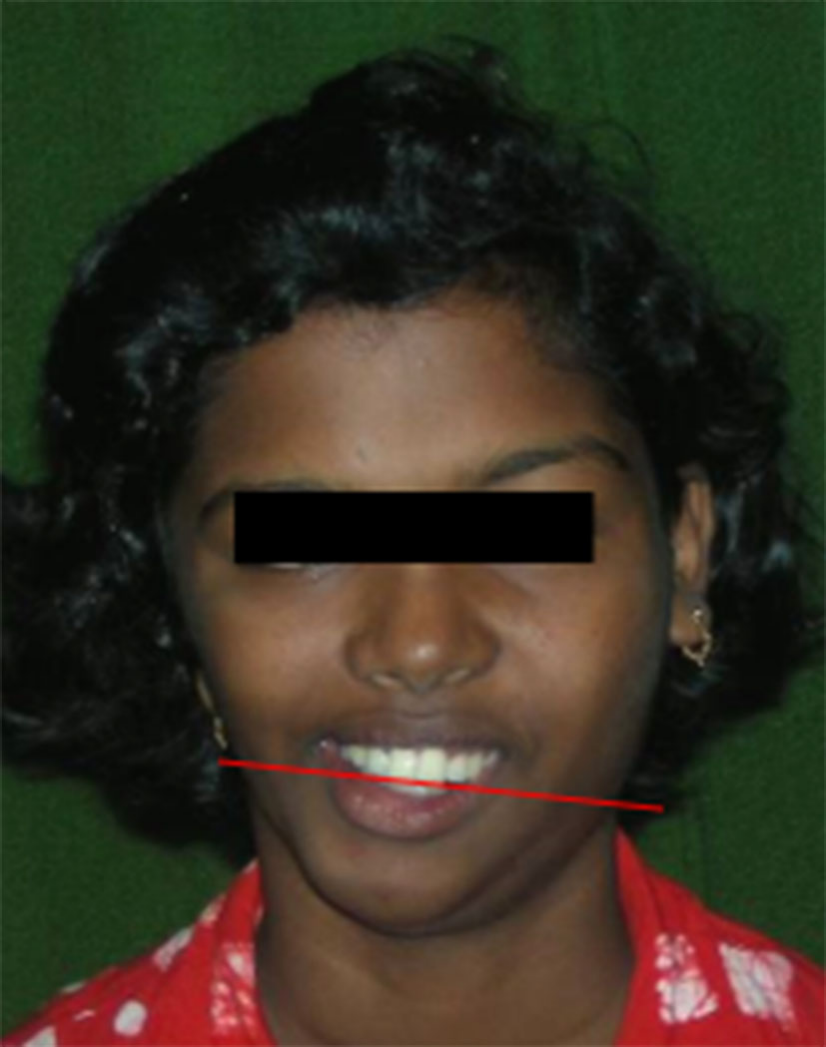
Cant of maxilla associated with Scoliosis

**Fig. 7:**
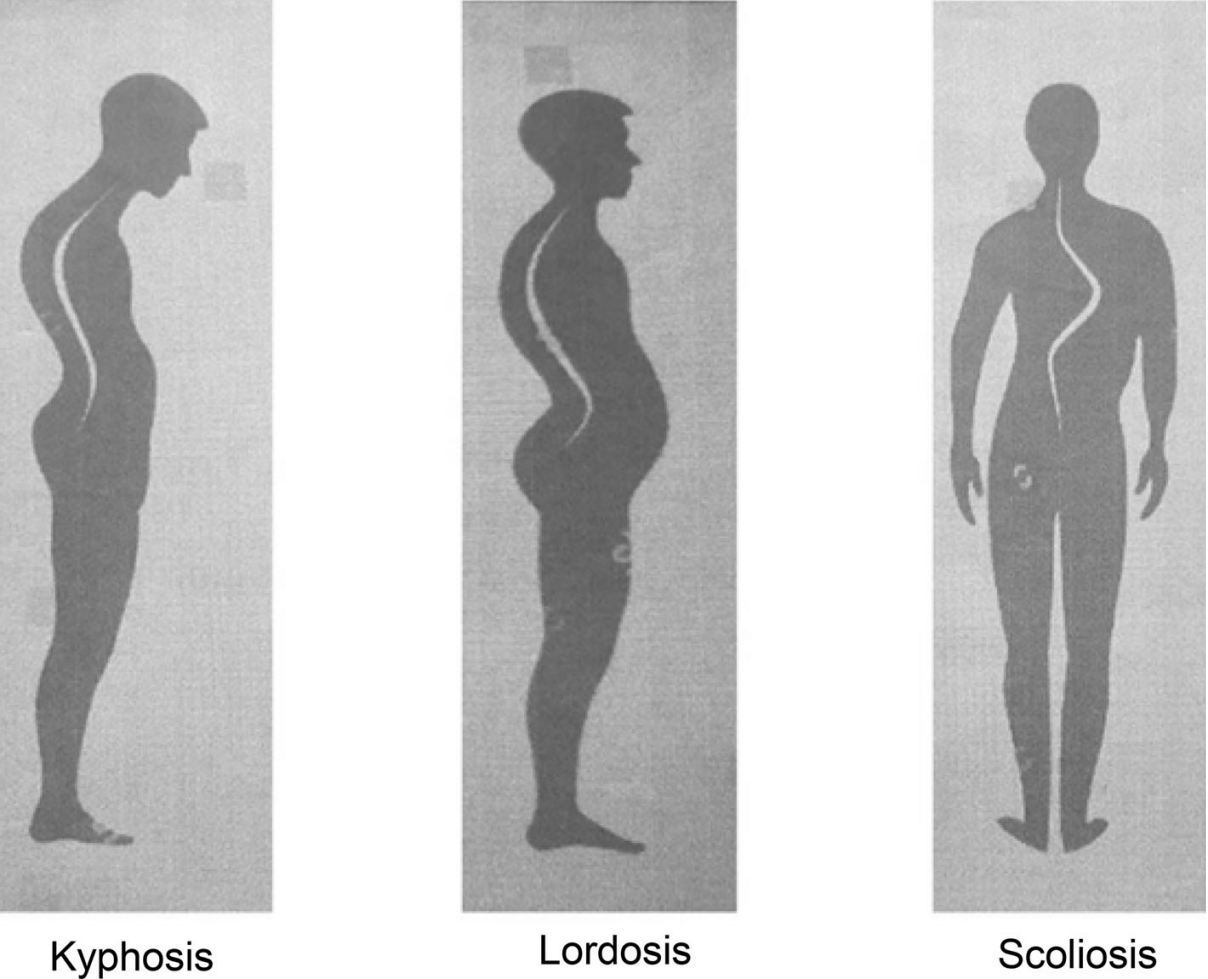
In both Class II and Class III and maxillary cant (scoliosis) situations spine is overloaded

**Fig. 8:**
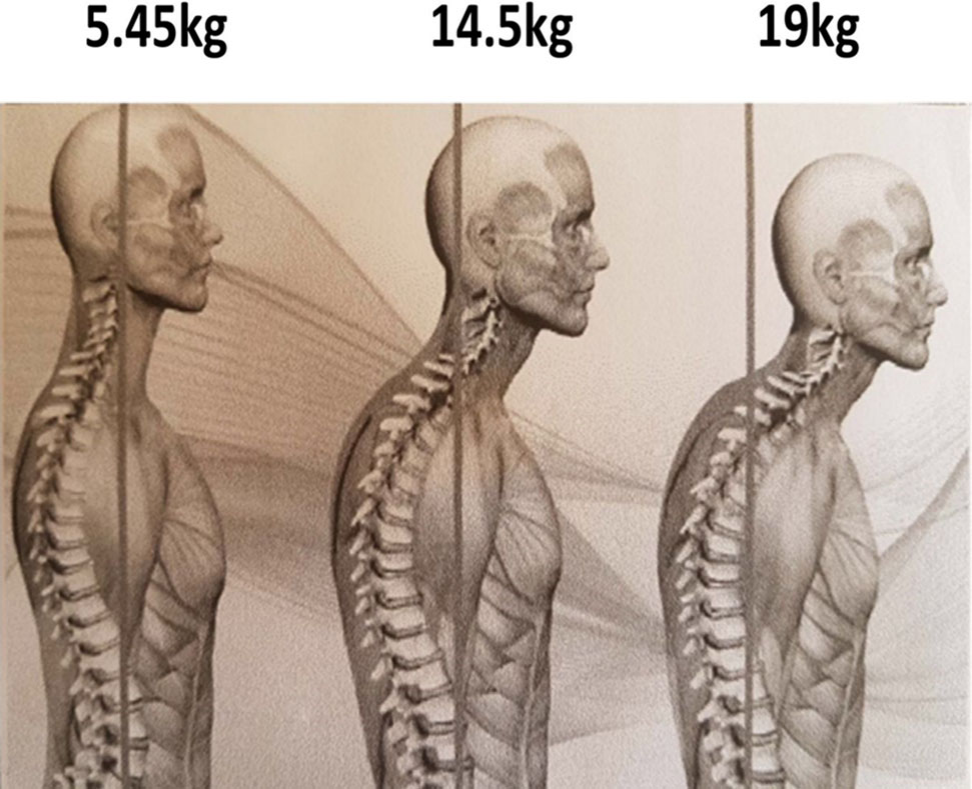
The over load generated can be anywhere between 14.5 and 19 k

Forward Head Posture (FHP) is a condition when the skull protrudes forwards more than an inch over the vertebra (atlas) in the neck on which the head rests. FHP is associated with anterior tilting of cervical spine. For every inch of forward movement of the head on the shoulders, the weight of the head increases by 4–5 k. That in turn leads to hyper actions of some muscles and weakening of others. Along with altered muscle actions, all the functions routed through spine get affected.

Diseases associated with overload on spine and bad posture are namely Headache, Jaw pain, Neck pain, Shoulder pain, Back pain, Hip pain, Knee pain, Ankle pain, Foot pain, Tinnitus, Watery eyes, Sinus pain, Chronic cough, Snoring, Bruxing, Impaired digestion, Ulcers, Weight gain, Hormonal imbalance and Sleep apnea.

According to American Academy of Sleep Medicine 26 percent of adults between the age of 30–70 years have Sleep apnea and according to American Sleep Association, it is estimated that 22 million Americans suffer from Sleep apnea with 80 percent of the cases of moderate and severe Obstructive Sleep Apnea Syndrome (OSAS) undiagnosed [7]. It is estimated in India 34 million people may be suffering from OSAS. Despite being a common disease, a large number of OSAS cases go undiagnosed due to lack of diagnostic facilities.

A case study of kyphotic posture, suffering from moderate OSAS, treated and normal airway restored.

Requisites:

- Clinical examination
- Face analysis
- Clinical pictures
- Lateral cephalogram analysis for airway analysis
- Orthopantomogram to visualize the position of condyle in the fossa
- Bite records (Fig. 9)

**Fig. 9:**
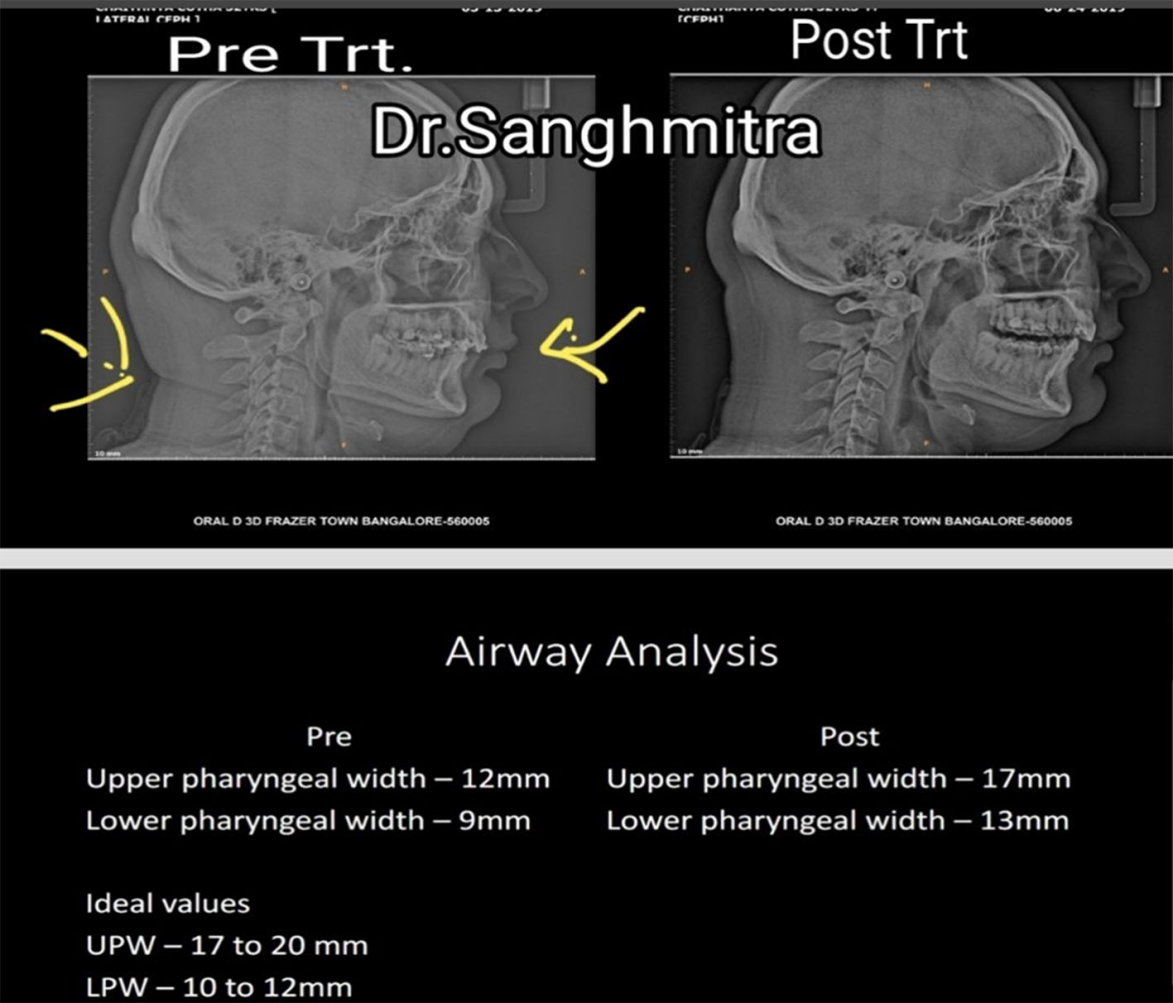
Pre and Post treatment cephalograms and Airway analysis values

**Table:** .

Pre treatment ceph shows	Post treatment ceph shows
1. Neck fold	1. Neck fold disappeared
2. Overloaded cervical spine	2. Cervical spine looks normal
3. Compromised airway	3. Normal airway
4. Reduced lower third of face	4. Normal lower third of face

For the normal growth of craniofacial structures, airway must be normal. Nasopharynx and Oropharynx both form a unit from which functions like respiration and deglutition happen. Significant relationship between the craniofacial, dentofacial and pharyngeal structures have been reported in several studies (Fig. 10).

**Fig. 10:**
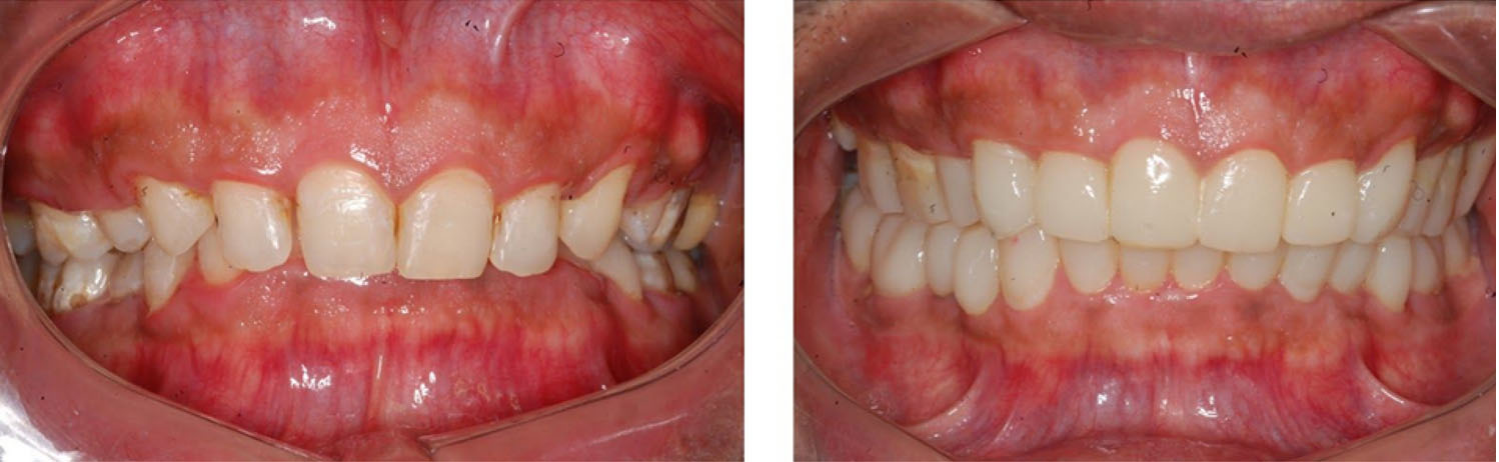
Pre treatment Post treatment

Normal upper pharyngeal space is 17–20 mm while lower pharyngeal airway space is 10–12 mm. When an individual breathes through the nose, the air is warmed, moistened, conditioned and mixed with nitric oxide. Deadly bacteria in the inhaled air are killed. Nitric oxide also works as vasodilator on the airways. Our body has a gene-T2R38 that stimulates receptors in the nose when air passes through. These receptors release nitric oxide which reacts with the chemicals that bacteria in the air use to communicate. The nitric oxide thus eliminates the bacteria resulting in the intake of relatively fresh air [8].

The vasodilation by nitric oxide increases the surface area of alveoli, where oxygen is absorbed in the bronchial tubes, which means more oxygen is absorbed when we breathe through our nose. Nasal breathing thus increases circulation, controls blood oxygen and carbon dioxide levels, slows the breathing rate and improves overall lung volumes [9].

The hypothalamus (Brains brain) is responsible for many functions in our bodies which we presume to be automatic. They are heartbeat, blood pressure, thirst, appetite and the cycles of sleeping and waking. The hypothalamus is also responsible for generating chemicals that influence memory and emotions.

The nasal cycle, which is part of an overall body cycle, is controlled by the hypothalamus. Sympathetic dominance on one side causes nasal vasoconstriction of the ipsilateral turbinate, while parasympathetic dominance in the other causes nasal vasoconstriction of the contralateral turbinate. Increased airflow through the right nostril is correlated to increased left brain activity and enhanced verbal performance. Increased airflow through the left nostril is associated with increased right brain activity and enhanced spatial performance [10].

The lungs are a primary source of our energy level. They extract oxygen from the air we breathe primarily during exhalation. When we exhale air through small nostrils compared to mouth, a back-pressure is created and exhaled air is restricted and slowed down, which is exactly the time lungs absorb more oxygen. It slows the air escape so the lungs have more time to extract oxygen from them. When there is proper oxygen-carbon dioxide exchange, the blood maintains a balanced pH. Our oxygen uptake happens mostly during the restricted process of exhalation through the nose. If carbon dioxide is lost too quickly, during mouth breathing, oxygen absorption is decreased [11].

Our nose is enabled to filter the air that we breathe. When we bypass nose and breathe through the mouth, there is nothing preventing the bad bacteria from gaining entry inside the body. One of the bacteria is Staphylococcus aureus which may cause diseases due to direct infection or produce toxins that could cause Staph infection in the heart and blood.

Our nasal passages have afferent stimuli—the nerves that regulate breathing. When inhaled air passes through the nose, nasal mucosa carries the stimuli to reflex nerves that control breathing. When we breathe through mouth we bypass nasal mucosa and it predisposes to loud snoring and irregular breathing. Snoring is a precursor to sleep apnea. Apnea is considered as a precursor to low cellular oxygen. This, in very severe cases, might lead to heart attacks or even death in one’s sleep.

Effect of reduced airway on breathing:

On an average

Total lung capacity 6 l

Tidal volume 500 ml. It is the amount of air taken in one stroke

Inspiratory reserve volume 3300 ml. It is the amount of air one can breath in apart from tidal volume

Expiratory reserve volume 1200 ml. It is the amount of air one can breath out apart from tidal volume

Reserve volume 1000 ml

An average male breathes 12 breath per minute, it is called minute ventilation

500 × 12 = 6000 ml of air per minute is received by the body. However 150 ml out of 500 ml remains unused for gaseous exchange called as dead space

That means only 350 ml of air is available to be utilized

350 × 12 = 4200 ml of air per minute is required at rest. This is called alveolar ventilation

Airway space of minimum 17 mm allows 4200 ml of air per minute

If the airway space reduces, say if it is only 12 mm (as in the above case), the air that an individual would be able to take in will be only 2964 ml per minute. That is a breathing inefficiency. Eventually to compensate, an individual breathes more than 12 times at rest. As a result brain instructs the heart lung machine to over work. This leads to increased blood pressure, increased heart beat and increased stress hormone cortisol in the blood stream leading to stress build up [12]. Restored airway space ensures normal minute or alveolar ventilation and reversal of onset of various ailments listed above.
